# Spontaneous splenic rupture and Anisakis appendicitis presenting as abdominal pain: a case report

**DOI:** 10.1186/1752-1947-6-114

**Published:** 2012-04-23

**Authors:** Joaquín Valle, Elisa Lopera, María Eugenia Sánchez, Rocío Lerma, Javier López Ruiz

**Affiliations:** 1 Department of Emergency Medicine, Hospital "Valle de los Pedroches," Calle Juan Del Rey Calero, S/N, Pozoblanco 14400, Córdoba, Spain; 2 Department of Pathology, Hospital "Valle de los Pedroches," Calle Juan Del Rey Calero, S/N, Pozoblanco 14400, Córdoba, Spain; 3 Department of Radiology, Hospital "Valle de los Pedroches," Calle Juan Del Rey Calero, S/N, Pozoblanco 14400, Córdoba, Spain; 4 Department of Surgery, Hospital "Valle de los Pedroches," Calle Juan Del Rey Calero, S/N, Pozoblanco 14400, Córdoba, Spain

## Abstract

**Introduction:**

Anisakidosis, human infection with nematodes of the family Anisakidae, is caused most commonly by *Anisakis simplex*. Acquired by the consumption of raw or undercooked marine fish or squid, anisakidosis occurs where such dietary customs are practiced, including Japan, the coastal regions of Europe and the United States. Rupture of the spleen is a relatively common complication of trauma and many systemic disorders affecting the reticuloendothelial system, including infections and neoplasias. A rare subtype of rupture occurring spontaneously and arising from a normal spleen has been recognized as a distinct clinicopathologic entity. Herein we discuss the case of a woman who presented to our institution with appendicitis secondary to *Anisakis *and spontaneous spleen rupture.

**Case presentation:**

We report the case of a 53-year-old Caucasian woman who presented with hemorrhagic shock and abdominal pain and was subsequently found to have spontaneous spleen rupture and appendicitis secondary to *Anisakis simplex*. She underwent open surgical resection of the splenic rupture and the appendicitis without any significant postoperative complications. Histopathologic examination revealed appendicitis secondary to *Anisakis simplex *and splenic rupture of undetermined etiology.

**Conclusions:**

To the best of our knowledge, this report is the first of a woman with the diagnosis of spontaneous spleen rupture and appendicitis secondary to *Anisakis simplex*. Digestive anisakiasis may present as an acute abdomen. Emergency physicians should know and consider this diagnosis in patients with ileitis or colitis, especially if an antecedent of raw or undercooked fish ingestion is present. Spontaneous rupture of the spleen is an extremely rare event. Increased awareness of this condition will enhance early diagnosis and effective treatment. Further research is required to identify the possible risk factors associated with spontaneous rupture of the spleen.

## Introduction

*Anisakis simplex *is a nematode belonging to the order Ascaridida, family Anisakidae and subfamily Ascaridoidea. Any fish or cephalopod species can be parasitized by third-stage *Anisakis *larvae. Codfish, hake, sardines, anchovies, salmon, tuna and squid are among the most frequently parasitized species. The ingestion of third-stage *Anisakis *larvae can cause anisakiasis in humans. Symptoms of anisakiasis arise when the nematode penetrates the gastric mucosa, which leads to serious abdominal and allergic symptoms [1]. In 1960, van Thiel *et al. *[2] reported the first published case of anisakiasis. Since then many cases have been reported in Japan and Western Europe, where raw fish are consumed frequently. The clinical manifestations depend on the effect of *Anisakis simplex *on the digestive tract wall. It is estimated that this entity is currently underdiagnosed, although published cases are becoming increasingly common. The presenting symptoms vary greatly, depending on where the larvae settle in the gastrointestinal tract. The infection can imitate several surgical conditions, such as ileus, appendicitis, peritonitis, ulcus and Crohn's disease [3]. Spontaneous rupture of the spleen is usually associated with infectious, neoplastic or hematologic diseases. Unlike traumatic splenic rupture, spontaneous rupture of the spleen is not often considered in the differential diagnosis of abdominal pain and can easily be confused with other abdominal pathology. Failure to include splenic rupture in the differential diagnosis can be catastrophic.

## Case presentation

A 53-year-old Caucasian woman was admitted to the emergency department (ED) at our institution because of sudden, severe abdominal pain after eating anchovies, as well as weakness, diaphoresis and hypotension. She rated her pain as 8 on a scale of 0 to 10 (with 10 representing the most severe pain). On examination, she was found to be pale, profusely diaphoretic and in extreme discomfort. At the time of admission, her blood pressure was 84/49 mmHg, her pulse was 110 beats/minute, her respiration was 20 breaths/minute and shallow and her oxygen saturation was 98% while she was breathing ambient air. Her neck was supple, and her chest was clear. Her abdomen was diffusely tender, with guarding in the epigastrium. The point of greatest tenderness was in the periumbilical area. Blood was drawn for laboratory testing, and a peripheral 20-gauge intravenous line was placed. Crystalloid intravenous solution was infused rapidly, and oxygen was administered at a rate of 2 L/minute by nasal cannula.

She had been evaluated several times previously in the ED because of abdominal pain. Her discomfort had begun 6 months earlier and was localized to the epigastrium. At that time, she described a constant pressure unrelated to food intake that was associated with intermittent nausea and vomiting. She reported no change in urine or stools and no hematochezia, melena, dysphagia, anorexia, increase in abdominal girth, early satiety or change in weight in the months before that admission. On examination at that time, her abdomen was slightly distended, her pain was improved, surgical etiologies were ruled out and she was discharged with close follow-up. Her medical history included hyperlipidemia, hypertension, gastroesophageal reflux disease and diabetes mellitus type 2. She had had no previous operations. She did not drink alcohol or use illicit drugs. She smoked cigarettes (20 pack-years). Her medications on admission included fluvastatin (80 mg daily), lansoprazol (15 mg daily), irbesartan (300 mg daily), metformin (850 mg twice daily) and acetylsalicylic acid (100 mg daily). The patient had had high serum-specific immunoglobulin E (IgE) (25 kU/L) against *A. simplex *in a previous analysis 4 months earlier. A previous upper endoscopy had revealed mild to moderate gastric edema of the greater curvature.

The patient had eaten anchovies 48 hours before the onset of symptoms, despite having been warned not to do so. Approximately 30 minutes after her admission to the ED and 60 minutes after the onset of symptoms, her blood pressure was 65/35 mmHg and her pulse was 118 beats/minute. She appeared to be in severe distress. Her abdomen was diffusely tender without abdominal ecchymosis or palpable masses. Distal pulses were present. Laboratory studies revealed that her white blood cell count was 13,160 cells/μl with predominant eosinophilia, hemoglobin concentration was 7.9 g/dl, serum C-reactive protein was > 67 U/L, D-dimer was 550 ng/mm, hematocrit was 22.4% with a mean corpuscular volume of 87.9 fl, platelet count was 301,000/mm^3^, sodium level was 138 mmol/L, potassium was 4.8 mmol/L, bicarbonate was 25 mmol/L, blood urea nitrogen was 44 mg/dl, creatinine was 0.6 mg/dl and glucose was 112 mg/dl. Her alanine aminotransferase level was 27 U/L, aspartate aminotransferase was 35 U/L, alkaline phosphatase was 45 U/L, total bilirubin was 0.5 mg/dl, total protein was 7.4 mg/dl and amylase was 47 U/L. Her international normalized ratio was 0.9. In addition to crystalloid intravenous solution, uncrossed packed red blood cells were transfused simultaneously.

The differential diagnosis of shock in this patient with abdominal pain included hemorrhage, which can result from a perforated viscus, a ruptured abdominal aortic aneurysm or a ruptured ovarian cyst. Processes that cause hypovolemia because of interstitial fluid sequestration, such as pancreatitis, or that cause relative hypovolemia, such as a sepsis syndrome due to bacterial peritonitis, were unlikely to have caused such precipitous decompensation. Cardiogenic causes were also considered.

**Figure 1:**
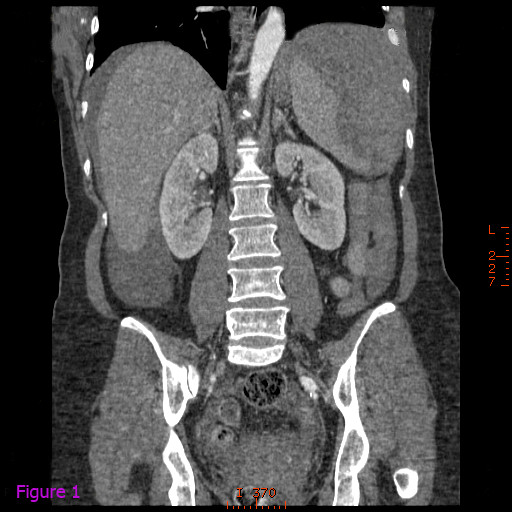
**Contrast-enhanced computed tomographic scan of the abdomen revealing splenic subcapsular hematoma with loss of normal structure and normal density of the lower pole, consistent with acute spontaneous splenic rupture**.

**Figure 2:**
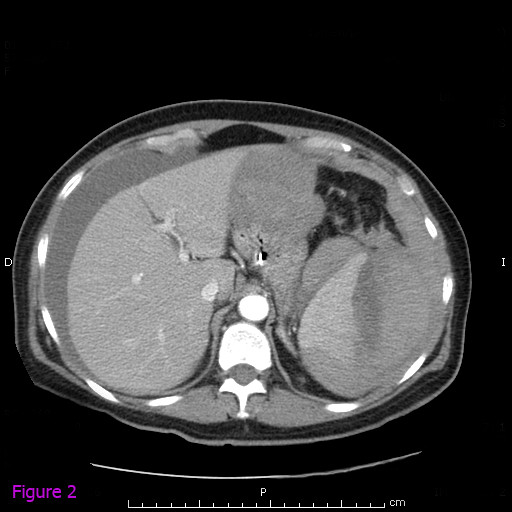
**Contrast-enhanced computed tomographic scout view of the abdomen showing splenic subcapsular hematoma with loss of normal structure and normal density of the lower pole, consistent with acute spontaneous splenic rupture**.

**Figure 3:**
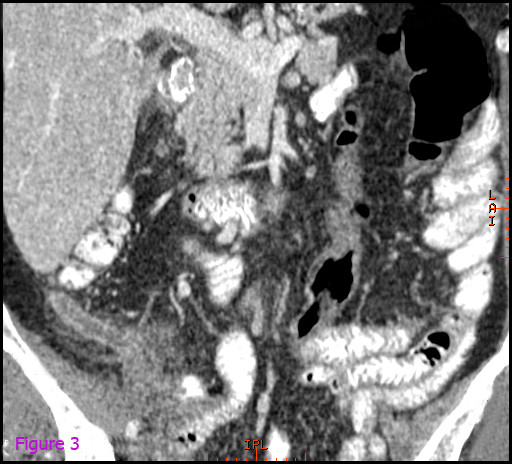
**Contrast-enhanced computed tomographic scan of the abdomen revealing the retrocecal region of the appendix with inflammation and mesenteric fat infiltration and a thickened appendiceal wall, suggesting acute appendicitis**.

**Figure 4:**
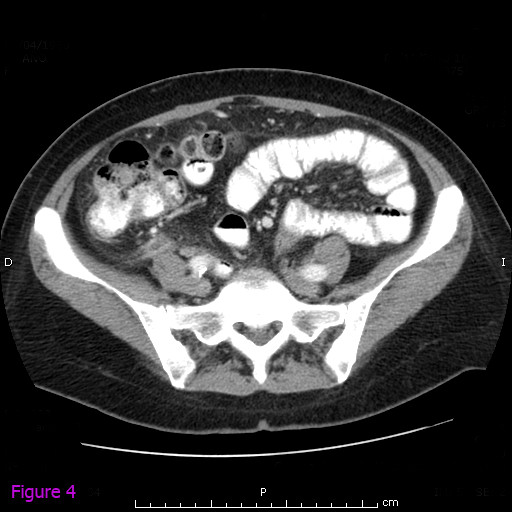
**Contrast-enhanced computed tomographic scout view of the abdomen revealing the retrocecal region of the appendix with inflammation and mesenteric fat infiltration and a thickened appendiceal wall, suggesting acute appendicitis**.

Contrast-enhanced computed tomographic (CT) scanning of the abdomen revealed a splenic subcapsular hematoma with loss of normal structure and normal density of the lower pole, consistent with acute spontaneous splenic rupture. It was accompanied by perihepatic, pelvic and perisplenic free fluid, compatible with hemoperitoneum (Figures 1 and 2). In the retrocecal region, the appendix showed inflammation and mesenteric fat infiltration. The appendiceal wall was thickened. These findings were suggestive of acute appendicitis (Figures 3 and 4). The diagnosis of anisakiasis was primarily suspected on the basis of common anamnesis (history of specific antibodies against *Anisakis *antigen in the serum), CT findings of appendicitis and the detection of eosinophilia in the differential blood picture.

**Figure 5:**
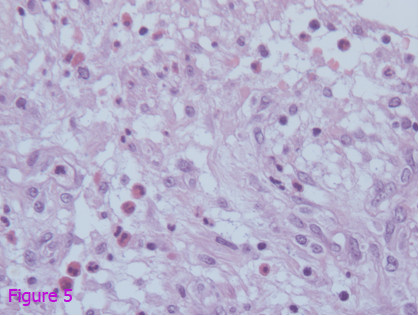
**Microscopic histopathological examination of the appendectomy specimen revealing the presence of intense inflammatory infiltrate composed of neutrophils and leukocytes accompanied by abundant eosinophils, suggestive of *Anisakis *appendicitis**.

**Figure 6:**
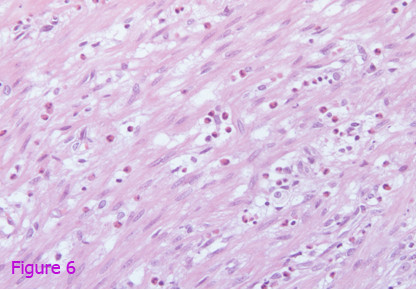
**Microscopic histopathological examination of the appendectomy specimen revealing the presence of intense inflammatory infiltrate composed of neutrophils and leukocytes accompanied by abundant eosinophils, suggestive of *Anisakis *appendicitis**.

After consultation with the surgeons, the patient was emergently taken to the operating room. Exploratory laparotomy was performed. After opening the abdomen, 2000 ml of blood were removed immediately. Surgery showed splenic subcapsular rupture and intraperitoneal blood with an otherwise normal spleen. The appendix was also removed, showing an acutely inflamed and non-perforated appendix. The pathology report demonstrated the spleen to be mildly enlarged and the parenchyma to be normal. The rupture was found on the medial side, penetrating into the parenchyma of the spleen. Histopathological analysis of the appendectomy specimen revealed the presence of intense inflammatory infiltrate composed of neutrophils and leukocytes accompanied by abundant eosinophils, suggestive of *Anisakis *appendicitis (Figures 5 and 6). Upon interrogation approximately 3 weeks later, the patient reported intermittent crampy abdominal pain located in the right lower quadrant that radiated to the right flank and was associated with borborygmi and diarrhea. The diagnosis was confirmed later by the detection of Anisakis Deoxyribonucleic acid (DNA) in the resected appendix on the basis of nested polymerase chain reaction. Paired acute-phase and convalescent sera provided no evidence of acute viral infection, and cultures from blood, sputum, stool and urine were negative. Screening for autoantibodies was negative. The patient's hospital course was uneventful, and she was discharged on postoperative day 5.

The results of serologic testing during the patient's hospital course and after discharge were negative throughout, including tests for antibodies to varicella zoster virus IgG. The results of all the following tests were negative: syphilis; antibodies to Epstein-Barr virus IgG and IgM, cryptococcus, rickettsia and human immunodeficiency virus (HIV); antigens of cytomegalovirus (CMV); and nucleic acids of CMV and HIV. The following test results were normal: direct Coombs antibody test; cold agglutinin screening and testing for lupus anticoagulant; hemoglobin electrophoresis; and levels of fibrinogen, homocysteine, lipoprotein (a), β_2_-glycoprotein I, anti-thrombin III and protein C (functional). At the time of her current admission her total IgE level was 760 kU/l and her specific IgE against *Anisakis simplex *was > 100 kU/l. Two months later her IgE level was 221 kU/l, and her specific IgE against *Anisakis simplex *was 34 kU/l. An upper endoscopy examination was later performed. Macroscopic examination revealed signs of moderate inflammation of the gastric mucosa, and a gastric biopsy revealed small areas of granulation tissue that had evidence of eosinophilic leukocyte infiltration within the gastric muscularis layer. The patient currently remains on active duty at work, and at her 18-month follow-up she reported doing well since being discharged, with no known sequelae.

## Discussion

Anisakiosis, previously called anisakiasis, is the formal term for the disease associated only with the genus *Anisakis *[4]. In Spain, it has been reported that the disease manifests as lesions in the small intestine in the majority of cases, but we are aware that the number of reported cases in Spain does not reflect the real frequency of anisakiosis [5]. Most symptoms associated with anisakiasis are due either to an allergic reaction or to direct tissue damage from *Anisakis *invasion of the gut wall [6]. Gastric anisakiasis usually develops from 1 to 8 hours after ingestion of raw fish, whereas enteric anisakiasis may take up to a few days to develop [7]. Gastric anisakiasis is characterized by acute epigastric pain, nausea and vomiting. Intestinal anisakiasis can lead to severe abdominal pain and an inflammatory reaction which may result in reactive intestinal obstruction [8]. Diarrhea with blood or mucus may also develop. Allergic reactions ranging from mild urticaria to anaphylactic shock can also occur [9,10]. Contact with inactivated *Anisakis simplex *material or proteins has also been described as producing hypersensitivity reactions, but this is still a matter of debate. Associated fever and peripheral eosinophilia are common. Although symptoms usually arise acutely, a chronic relapsing course can sometimes occur. These symptoms may mimic seafood allergy.

Complications include abdominal distention or obstruction related to the inflammatory mass around the parasite. The mass may be palpable on abdominal examination. Eosinophilic gastroenteritis or enterocolitis can also occur, and a syndrome that mimics appendicitis may be seen if the ileocecal region is involved [11,12]. Because the symptoms are vague, this disease is often misdiagnosed as appendicitis, acute abdomen, stomach ulcer or ileitis. *Anisakis *larvae occasionally penetrate into the peritoneal cavity or other visceral organs (extraintestinal anisakiasis) and cause eosinophilic granuloma, which may be confused with neoplasm [8]. The diagnosis of anisakiasis can be made in several ways: (1) by endoscopic examination, which may reveal an ulcerated bleeding lesion within the stomach or duodenum, at the center of which may be a worm measuring approximately 2 to 2.5 cm by 1 to 2 mm; (2) by barium studies, which may show narrowing of the intestinal lumen in areas with mucosal inflammation, a thread-like filling defect suggestive of a worm on imaging studies in some cases [13]; and (3) serial measurement of specific and total IgE when it is not known if the presence of specific IgE is due to a past infection or is associated with the present case [14].

Spontaneous rupture of the spleen is extremely rare [15-17], a small group of cases exist, in which the only justifiable conclusion is that the spleen ruptures spontaneously without known cause. Orloff and Peksin [18] identified four criteria for the diagnosis of spontaneous splenic rupture: (1) upon thorough questioning, either prior to surgery or in retrospect after surgery, the patient should reveal no history of trauma or unusual activity that could conceivably have injured the spleen; (2) there should be no evidence of disease in other organs that are known to affect the spleen adversely and thereby cause it to rupture; (3) there should be no evidence of perisplenic adhesions or scarring of the spleen that suggests it has been traumatized or ruptured previously; and (4) without findings of hemorrhage and rupture, the spleen should be normal on both gross inspection and histological examination. Crate and Payne [19] added a fifth criterion: studies of acute-phase and convalescent sera should not show any significant rise in viral antibody titers that are suggestive of recent infection with viruses associated with splenic involvement. The patient in our case, who was admitted with spontaneous splenic rupture, met these five criteria.

The pathophysiology of spontaneous splenic rupture is obscure. It should be considered that undetected structural abnormalities within the spleen may cause nontraumatic splenic rupture. Pathological rupture of the spleen is most commonly seen in the hematological malignancies [20], in which fragmentation and dissolution of the fibrous capsule of the spleen occur by infiltrating atypical lymphocytes, as seen in lymphoma or leukemia [21]. The most common symptom is left upper-quadrant abdominal pain. This pain can become generalized, with distention, tenderness and rigidity occurring in later stages. The abdominal symptoms may be accompanied by pallor, tachycardia, hypotension and oliguria. Eventually, more than half of patients will go into hemorrhagic shock if the condition is left untreated. The diagnosis is based on clinical symptoms and confirmatory diagnostic tests.

Four clinical features define the systemic inflammatory response syndrome (SIRS): fever or hypothermia, tachycardia, tachypnea and leukocytosis or leukopenia [22]. The patient described in our present report fulfilled three of these four criteria: tachycardia, tachypnea and leukocytosis. The presence of SIRS conjures a differential diagnosis that includes non-infectious and infectious causes. We can use our understanding of the pathophysiology of this overwhelming and dysregulated inflammatory response as a framework for our differential diagnosis. Two different pathways may result in SIRS [23,24]. One is triggered by the release of endogenous cellular contents from damaged tissue or dying cells, known as "damage-associated molecular patterns" (DAMPs). The other is triggered by exogenous microbial exposure: so-called "pathogen-associated molecular patterns" (PAMPs). Both the endogenous DAMPs and the exogenous PAMPs are recognized by pattern recognition receptors on cells of the innate immune system, including polymorphonuclear leukocytes and macrophages. Having ruled out endogenous causes of SIRS, we are left with the far more likely possibility that SIRS in our patient was triggered by an exogenous microbial PAMP. We believe that subacute appendicitis triggered a systemic inflammatory response, producing systemic inflammation particularly involving the spleen and causing spontaneous rupture.

## Conclusions

Anisakiasis is an increasingly frequent disease worldwide, as increased mixtures of different cultures and international traveling allow the spread of risky eating habits. Anisakiasis should be considered in the differential diagnosis of acute abdomen in patients with positive anamnesis for suspiciously cooked seafood eating. Spontaneous splenic rupture is a rare entity that needs a high index of suspicion for its diagnosis. The absence of a history of trauma can make it difficult to reach a diagnosis, which causes delay in treatment. Rapid diagnosis, aggressive resuscitation and surgical intervention can lead to successful outcomes in patients with spontaneous splenic rupture. Our present case highlights that spontaneous splenic rupture can occur with no obvious cause in a previously well, hemodynamically stable patient and should be suspected in patients with non-specific abdominal pain.

## Consent

Written informed consent was obtained from the patient for publication of this case report and any accompanying images. A copy of the written consent is available for review by the Editor-in-Chief of this journal.

## Competing interests

The authors declare that they have no competing interests.

## Authors' contributions

JVA was the emergency physician on duty who admitted the patient. He also contributed to the analysis and interpretation of the data and wrote the manuscript. EL made substantial contributions to the drafting of the manuscript and revised it. MES was the pathologist who interpreted the pathology slide. RL was the consultant radiologist who analyzed the case history and computed tomographic scans. JLR was the chief surgeon and was involved in drafting the manuscript and critically revising it for important intellectual content. All authors read and approved the final manuscript, and all authors contributed equally to the final draft of the manuscript.
